# Real life use of ravulizumab in Italian patients with paroxysmal nocturnal hemoglobinuria: evidence from the REACTION observational study

**DOI:** 10.1007/s00277-026-06792-w

**Published:** 2026-01-22

**Authors:** Anna Paola Iori, Antonio De Vivo, Eros Di Bona, Giovanni Caocci, Francesca Fioritoni, Fabio Ciceri, Eloise Beggiato, Davide Rapezzi, Angela Amendola, Amalia Figuera, Carmine Selleri, Francesco Longu, Bruno Fattizzo, Alessandra Tucci, Alessandro Cignetti, Valeria Amico, Simona Sica, Elisabetta Metafuni, Simona Raso, Tiziana Anna Urbano, Luana Marano, Nicola Di Renzo, Pierangelo Spedini, Alessandro Rambaldi, Francesco Lanza, Cristina Clissa, Cristina Danesin, Maria Bruna Greve, Sergio Cabibbo, Alessandra Ori, Francesca Cassanelli, Federica Sottana, Benedetta Campolo, Giulia Gasparri, Fabio Carini, Wilma Barcellini

**Affiliations:** 1 A.O.U. Policlinico Umberto I-Ematologia, Rome, Italy; 2 A.O.U. Policlinico Sant’Orsola-UOC Ematologia, Bologna, Italy; 3 Ospedale San Bassiano-Oncoematologia, Bassano del Grappa (Vicenza), Italy; 4 Ospedale Businco-Ematologia e CTMO, Cagliari, Italy; 5 Ospedale Santo Spirito-UOC Ematologia, Pescara, Italy; 6 Università Vita-Salute San Raffaele, Milan, Italy; 7 IRCCS Ospedale San Raffaele, Milan, Italy; 8 A.O.U. Città della Salute e della Scienza-Ematologia, Turin, Italy; 9 ASO Santa Croce e Carle-Ematologia, Cuneo, Italy; 10 AOR SAN CARLO, Dipartimento Oncologico, UOC Ematologia con Centro trapianto di Midollo, Cellule staminali e terapie cellulari, Potenza, Italy; 11 A.O.U. Policlinico Rodolico-San Marco-Ematologia, Catania, Italy; 12 A.O.U. S.Giovanni di Dio e R.D’Aragona-UOC Ematologia e Trapianti Cellule Staminali Emopoietiche, Salerno, Italy; 13 A.O.U. Sassari-Ematologia, Sassari, Italy; 14 Fondazione IRCCS Ca’ Granda Ospedale Maggiore Policlinico-SC Ematologia, Milan, Italy; 15 Department of Oncology and Hemato-Oncology, University of Milan, Milan, Italy; 16 ASST Spedali Civili-Ematologia, Brescia, Italy; 17 A.O. Ordine Mauriziano-SCDU Ematologia e Terapie cellulari, Turin, Italy; 18 Azienda Ospedaliera San Pio-Servizio di Immunoematologia, Benevento, Italy; 19 Fondazione Policlinico Universitario Agostino Gemelli IRCCS-UOC Ematologia e Trapianto di cellule staminali emopoietiche, Università Cattolica Sacro Cuore, Rome, Italy; 20 Fondazione Policlinico Universitario Agostino Gemelli IRCCS-UOC Ematologia e Trapianto di cellule staminali emopoietiche, Rome, Italy; 21 A.O.O.R. Villa Sofia Cervello P.O. Cervello-UOC di Ematologia per le Malattie Rare del Sangue e degli Organi Ematopoietici, Palermo, Italy; 22 Ospedale San G. Moscati-SC Ematologia, Taranto, Italy; 23 Ospedale San G. Moscati-Ematologia, Avellino, Italy; 24 Ospedale V. Fazzi-UOC Ematologia, Lecce, Italy; 25 Ospedale di Cremona-Ematologia, Cremona, Italy; 26 ASST Papa Giovanni XXIII-UOC Ematologia, Bergamo, Italy; 27 Università di Bologna - Ospedale S. Maria delle Croci-UOC Ematologia, Ravenna, Italy; 28 A.O.U.I. Verona Ospedale Borgo Roma-UOC Ematologia e Centro Trapianti di Midollo Osseo, Verona, Italy; 29 Ospedale Ca’ Foncello-Ematologia, Treviso, Italy; 30 GOM Bianchi Melacrino Morelli-Ematologia, Reggio Calabria, Italy; 31 Ospedale “Giovanni Paolo II”-UOSD Ematologia, Ragusa, Italy; 32 IQVIA Solutions Italy S.r.l., Modena, Italy; 33 Alexion Pharma Italy, Milan, Italy; 34 Alexion Pharma, Barcelona, Spain; 35 Fondazione IRCCS Ca’ Granda Ospedale Maggiore Policlinico-SC Ematologia, Milan, Italy

**Keywords:** Paroxysmal nocturnal hemoglobinuria, Ravulizumab, Lactate dehydrogenase, Breakthrough hemolysis, Health-related quality of life

## Abstract

Ravulizumab is a second-generation C5i engineered from eculizumab to achieve immediate, complete, and sustained inhibition of terminal complement activity in PNH. The REACTION observational cohort study describes the effectiveness and tolerability of ravulizumab in Italian patients who were previously treated with eculizumab. Eighty-one PNH patients were enrolled in this study. The primary endpoint was the percentage change in lactate dehydrogenase (LDH) from baseline to the end of observation (52 weeks follow-up). Among secondary endpoints, transfusion avoidance, breakthrough hemolysis (BTH) and patients’ quality of life (QoL) were evaluated. The median (25–75 percentiles) percentage change in LDH at 52 weeks follow-up was -2.6 (-11.5–13.4) U/L, with 92.3% of the patients presenting LDH within or < 1.5 × upper limit of normal (ULN). Overall, 20 (25.0%) patients required transfusion during the eculizumab period and 15 (18.8%) during the ravulizumab. Seven BTH events were observed, 5 during eculizumab period and 2 (triggered by other medical conditions) during ravulizumab, suggesting the reduction of pharmacokinetic BTH during ravulizumab treatment. EORTC-QLQ-C30 and FACIT-Fatigue scores were similar to the general population, and patients’ preference indicated ravulizumab as the favorite treatment. The REACTION study confirmed the effectiveness of ravulizumab in maintaining stable disease and hemolysis control in the real-world setting. Clinical trial registration. NCT05274633, 02-Mar-2022.

**Supplementary Information:**

The online version contains supplementary material available at 10.1007/s00277-026-06792-w.

## Introduction

Paroxysmal nocturnal hemoglobinuria (PNH) is a rare, chronic, potentially life-threatening hematological disorder caused by uncontrolled terminal complement activation of blood cells and is associated with intravascular hemolysis, thromboembolic events, organ damage, impaired quality of life and increased mortality. PNH is caused by somatic mutations in the phosphatidylinositol glycan anchor biosynthesis class A (PIGA) gene of bone marrow stem cells. All cell lineages of bone marrow stem cells are affected including red blood cells, leucocytes, and platelets [1–4].

The worldwide PNH estimated incidence ranges from 1 to 1.5 cases per million people to 3.81 per 100,000 individuals per year [2, 5]. In untreated PNH, the median survival is 10–20 years, with thrombosis as the leading cause of death, accounting for approximately 40–67% of deaths with known causality. Moreover, the mortality risk is ≥ 4-fold higher in patients with a history of thromboembolic events [2, 5–7]. The clinical manifestations of PNH result from hemolysis driven by complement activation in unprotected red cells, leukocytes, and platelets, and from the release of free hemoglobin into the bloodstream that occurs with erythrocyte destruction [8].

The primary symptoms of PNH are anemia, jaundice and hemoglobinuria/hemosiderinuria (reported in almost 50% of cases), and fatigue, most intense during a hemolytic attack but usually present at all times (80.9% of patients in the International PNH Registry reported fatigue) [2, 3, 5, 6]. Additionally, due to the toxic effect of the free hemoglobin in the bloodstream, patients could present with progressive renal damage, which can culminate in renal failure due to the buildup of hemoglobin deposits in the kidney, and with smooth muscle spasms and ischemia caused by the local nitric oxide depletion and resulting in dysphagia, chest pain, abdominal pain, dyspnea, pulmonary hypertension, increased risk of thrombosis, and erectile dysfunction [6, 8].

C5 inhibitors (C5i), eculizumab and ravulizumab, the current standard of care for PNH, inhibit terminal complement activation to prevent intravascular hemolysis and thrombosis, the disease processes that drive PNH morbidity and mortality [6, 9]. Eculizumab was the first approved treatment for patients with PNH and was authorised in Italy in 2007; ravulizumab, a second-generation C5i, was authorised in Italy in July 2019. At the time of study initiation these drugs were the only reimbursed treatments for PNH.

Eculizumab, a humanized monoclonal antibody that inhibits terminal complement C5 activation, was the first approved treatment for patients with PNH and has changed the paradigm of PNH management [2].

Intravenous infusion of eculizumab every 2 weeks reduces hemolysis, anemia and occurrence of thrombosis and increases hemoglobin stabilization, improving the rate of transfusion independence and enhancing patient quality of life (QoL) [3, 4, 6]. Despite its established efficacy, up to 27% of patients still experience breakthrough hemolysis (BTH) while on approved doses of eculizumab. This issue may be linked to suboptimal exposure due to the drug’s pharmacokinetic profile, sometimes requiring shorter dosing intervals of less than 14 days or higher individual dosages. Also, patients may experience low-level extravascular hemolysis sometimes requiring transfusions [5, 10]. In addition, the eculizumab dosing regimen requiring IV infusion every two weeks may have a negative impact on the patient’s quality of life [10].

Ravulizumab is a second-generation C5i, engineered from eculizumab, with a longer half-life than eculizumab, which allows for a longer period between infusions (every 8 weeks instead of every 2 weeks) due to a sustained inhibition of C5 [2, 6, 9, 11]. Ravulizumab showed non-inferiority compared with eculizumab in both adult patients with PNH naïve to complement inhibitors and in adult patients with PNH who had previously been treated with a C5 inhibitor [2, 7, 10, 12–14]. The longer half-life of ravulizumab, with its immediate, complete and sustained inhibition on terminal complement activity along with the convenience of frequency of administration significantly improved patient QoL and significantly reduced pharmacokinetic BTH events [2].

Long-term data from registrational clinical trials of ravulizumab, including both C5-inhibitor-naive patients and those with previous experience with eculizumab, indicated that few BTH events were reported with ravulizumab. These events were generally linked to complement-amplifying conditions. Moreover, only a small percentage of BTH events (1.8%, *n* = 2) were associated with suboptimal inhibition of C5 [7]. The safety profiles of eculizumab and ravulizumab were similar, with the overlapping frequency of headaches (up to one-third in the first period) and a small but clinically significant risk of meningococcal infection [14].

New long-term real-world data are necessary to describe the effectiveness and tolerability of a switch from eculizumab to ravulizumab in patients with stable disease and assess their impact on QoL and patient preferences among the two treatments. The REACTION study, a multi-center observational cohort study, aims to assess the effectiveness and tolerability of ravulizumab in Italian patients with PNH who have switched to ravulizumab after at least 26 weeks of treatment with eculizumab.

## Materials and methods

### Study design and patient population

The REACTION study is an Italian multicenter, observational, non-interventional cohort study composed of both retrospective and prospective observation periods on the same PNH patients. Patients were enrolled between 2022 and 2023. Baseline was defined as the start of ravulizumab treatment after the switch from eculizumab, and patients could be enrolled at or after baseline. The prospective observation window (enrolment; + 52 weeks) corresponded to the period of treatment with ravulizumab; while the retrospective observation window corresponded to the period of treatment with eculizumab (baseline; -52 weeks) or, if enrolment took place after the baseline, to the retrospective period of treatment with ravulizumab plus the period with eculizumab. The end of observation was defined as the achievement of 52 weeks (± 4 weeks) follow-up during treatment with ravulizumab or patient withdrawal.

The main inclusion criteria were: (a) adult patients (≥ 18 years) with documented diagnoses of PNH confirmed by high-sensitivity flow cytometry evaluation of red blood cells and white blood cells with granulocyte or monocyte clone size of ≥ 5%; (b) treated with eculizumab for at least 26 weeks; (c) already assigned to ravulizumab treatment as specific therapeutic strategy within current routine clinical practice before the enrolment of the patient in the study; (d) vaccinated against Neisseria meningitidis (according to SmPC) < 3 years before dosing or at the time of study drug initiation to reduce the risk of meningococcal infections; (e) subject informed consent and privacy form signature prior to study participation.

Patients with a history of hematopoietic stem cell transplantation, known pregnant or breastfeeding patients, unable to read and write in Italian language and to autonomously fill in questionnaires and scales, and patients enrolled in any clinical trial receiving experimental treatments for PNH were excluded.

## Study objectives

The primary endpoint of the study was the percentage change in lactate dehydrogenase (LDH) from baseline to the end of observation during ravulizumab treatment in standard clinical practice in Italy.

The secondary endpoints were: (a) the percentage change in LDH from baseline to end of observation in patients treated with ravulizumab with respect to the observed treatment period with eculizumab; (b) the proportion of patients who needed transfusions during the treatment period with ravulizumab with respect to the observed treatment period with eculizumab; (c) the proportion of patients undergoing ravulizumab without a ≥ 2-g/dL decrease in hemoglobin level in the absence of transfusion, in the ravulizumab and eculizumab treatment period; and (d) the proportion of patients experiencing BTH during both ravulizumab and eculizumab treatment. BTH was defined as at least one new or worsening symptom or sign of intravascular hemolysis (fatigue, hemoglobinuria, abdominal pain, shortness of breath [dyspnea], anemia [hemoglobin < 10 g/dL], major adverse vascular event including thrombosis, dysphagia, or erectile dysfunction) in the presence of elevated LDH ≥ 2 × upper limit of normal (ULN) after prior LDH reduction to < 1.5 × ULN while on therapy.

Additionally, secondary endpoints were: (a) QoL during ravulizumab treatment in the routine clinical practice, evaluated using the Functional Assessment of Chronic Illness Therapy (FACIT)-Fatigue scale and the European Organisation for Research and Treatment of Cancer (EORTC) QLQ-C30 scale, (b) the patient’s preference on treatment, evaluated according to the PNH-specific Patient Preference Questionnaire (PNH-PPQ) at the end of the observation period.

The safety profile was evaluated during the prospective period of treatment with ravulizumab.

## Data source

The REACTION study involved both primary and secondary data collection: (a) primary data collection was performed during the prospective observation period or until the patient’s early withdrawal; (b) secondary data collection was performed during the retrospective observation period, from enrolment backward to eculizumab initiation date (52 weeks as maximum, in case of earlier treatment initiation).

Data regarding the whole observation were retrieved from the hospital medical charts of participant sites according to their ordinary clinical practice or from other documents in accordance with Italian regulations. The collected data were entered into an electronic Case Report Form (eCRF).

### Statistical analysis

The sample size was not based on statistical considerations but rather was defined considering the extremely rare characterization of PNH and the number of potentially eligible patients in the selected sites (clinical judgment). It was considered a target sample of about 120 patients, with a 20% drop-out, expecting around 96 patients to be available for the evaluation.

No formal hypotheses were set for this observational study, which had a descriptive aim. Descriptive statistics (e.g. mean (standard deviation, SD), median (25th – 75th percentiles [P]) have been reported.

The safety profile of ravulizumab was described through descriptive statistics, reporting the number and proportion of patients with adverse events (AE) and serious adverse events (SAE) together with the total number of such events.

The analyses were performed on the Full Analysis Set, including the patients meeting all inclusion and none of the exclusion criteria and on the Safety Set, composed of all the enrolled patients who started the treatment with ravulizumab and signed the informed consent form.

SAS Enterprise Guide 8.2 and SAS for Windows Version 9.4 was used for statistical analyses.

IQVIA Solutions Italy (formerly Medineos) supported in the design, conduct and statistical analysis of the REACTION study.

## Results

A total of 81 patients were enrolled in the REACTION study across 28 Italian centres: 80 met the eligibility criteria and were included in the Full Analysis Set, and all 81 were included in the Safety Set. Median [25th – 75th P] observation period was 50.0 (50.0–50.7) weeks. A total of 9 patient prematurely withdrawn from the study due to: enrolment in any clinical trial on experimental treatments for PNH (3; 33.3%), loss to follow-up (2; 22.2%), not-treatment-related death (2; 22.2%), interruption of ravulizumab (2; 22.2%).

Patient demographics and baseline clinical characteristics are described in Table 1. As for the inclusion criteria, the cohort was represented by adult patients diagnosed with PNH (median [25th – 75th P] time of 9.0 [4.3–16.7] years), previously treated with eculizumab. Eculizumab was administered at 900 mg every 14 days for a median (25th – 75th P) time of 6.1 (2.9–10.7) years.

**Table 1:** Demographics and clinical characteristics of the eligible patients

	Full set analysis (*N* = 80)
Age at index date, mean (SD)	50.5 (16.1)
Gender, n (%)	
Male	39 (48.8%)
Female	41 (51.3%)
Race/Ethnicity#, n (%)	
Asian	4 (5.7%)
Black	1 (1.4%)
White/Caucasian	65 (92.9%)
BMI classes at index date#, n (%)	
Underweight	2 (3.1%)
Normal weight	29 (44.6%)
Overweight	25 (38.5%)
Obese	9 (13.8%)
UNK	15
Years since PNH diagnosis - median (25th – 75th P)	9.0 (4.3–16.7)
PNH associated with bone marrow disease, n (%)	
Yes	25 (31.3%)
Conditions at baseline§, n (%)	
None	52 (65.0%)
Aplastic or hypoplastic anemia	19 (23.8%)
Myelodysplastic syndrome	5 (6.3%)
Bone marrow disorder	1 (1.3%)
Other	4 (5.0%)
Ongoing symptoms at baseline§, n (%)	
None	55 (68.8%)
Fatigue	19 (23.8%)
Hemoglobinuria	6 (7.5%)
Dyspnea	2 (2.5%)
Abdominal pain	1 (1.3%)
Dysphagia	1 (1.3%)
Other	3 (3.8%)

BMI = Body Mass Index; PNH = Paroxysmal Nocturnal Hemoglobinuria; P = Percentile; SD = Standard Deviation; UNK = Unknown

# Percentages computed excluding patients with “UNK” and missing data from the total

§ More than one option could have been recorded

Overall, 41 (51.3%) subjects were females, 65 (92.9%) were White/Caucasian, and the mean (SD) age was 50.5 (16.1) years. At baseline, 31.4% of the patients presented with aplastic or hypoplastic anemia, myelodysplastic syndrome and/or bone marrow disorder, most patients (52; 65.0%) had no comorbidities, and most patients (55; 68.8%) had no PNH symptoms, with fatigue (19; 23.8%) the most commonly reported.

At baseline, patients switched to ravulizumab treatment, administered every 8 weeks according to its recommended dosing regimen based on the patient’s body weight, consisting of an induction dose of 2700.0 mg/mL for 58 (72.5%) patients followed by maintenance dosing of 3300.0 mg/mL for 57 (71.3%) patients. The median (25th – 75th P) treatment duration with ravulizumab was 50.0 (50.0–50.7) weeks. Two patients discontinued treatment during observation, one discontinuation was permanent due to change of therapy. No patient switched back to eculizumab during observation.

At the end of the observation period (52-week follow-up visit), the median (25th – 75th P) percentage change in LDH from baseline was -2.6 (-11.5–13.4) (primary endpoint). The change was consistent during the study observation period, showing hemolysis control during the eculizumab treatment period and after the switch to ravulizumab (Table 2). In particular: at baseline (eculizumab treatment period), the median (25th – 75th P) LDH value was 248.5 (203.0–306.0) U/L, 89.1% (*n* = 66) of the patients presented with LDH within the ULN or < 1.5 × ULN, and the median (25th – 75th P) hemoglobin level was 10.8 (9.5–11.9) g/dL. At the 52-week follow-up visit (ravulizumab treatment period), the median (25th – 75th P) LDH value was 251.0 (204.0–310.0) U/L, 92.3% (*n* = 60) of the patients presented with LDH within the ULN or < 1.5 × ULN, and the median (25th – 75th P) hemoglobin level was 11.2 (9.9–12.5) g/dL.

**Table 2:** Percentage change in LDH from baseline to the 52-week follow-up visit (primary endpoint) and during the observation period (secondary endpoint)

	*N*	Median (25th – 75th) *P*
Percentage change in LDH value from 52 weeks prior baseline to baseline	57	-0.6 (-10.0–9.7)
LDH value (U/L) at 52 weeks prior baseline	59	253.0 (204.0–312.0)
Percentage change in LDH value from 26 weeks prior baseline to baseline	62	-0.7 (-7.9–11.2)
LDH value (U/L) at 26 weeks prior baseline	63	238.0 (205.0–286.0)
Percentage change in LDH value from baseline to 18 weeks follow-up	63	1.2 (-11.1–12.2)
LDH value (U/L) at 18 weeks follow-up	66	250.0 (208.0–296.0)
Percentage change in LDH value from baseline to 34 weeks follow-up	63	2.7 (-8.1–15.4)
LDH value (U/L) at 34 weeks follow-up	66	260.0 (202.0–307.0)
Percentage change in LDH value from baseline to 52 weeks follow-up	62	-2.6 (-11.5–13.4)
LDH value (U/L) at 52 weeks follow-up	65	251.0 (204.0–310.0)

*LDH = *Lactate dehydrogenase; *P = *Percentile

The transfusion need corresponded to 5.4 units per patient during eculizumab treatment period and 4.6 units per patient during ravulizumab treatment. Specifically, during the eculizumab treatment period, 25.0% (*n* = 20) of the patients received in total 108 packed red blood cell units, while in the ravulizumab treatment period, 18.8% (*n* = 15) of the patients received in total 69 packed red blood cell units. Five patients required transfusions while treated with eculizumab and were transfusion-free after the switch to ravulizumab. Seven patients receiving transfusion during eculizumab treatment had bone marrow disease at baseline. The median (25th – 75th P) hemoglobin level before transfusion was 7.9 (7.3–8.3) g/dL during the eculizumab treatment period and 7.7 (6.7–8.2) g/dL during the ravulizumab treatment period. The main reasons for transfusion were intravascular and extravascular hemolysis (61.3% [*n* = 54] during the eculizumab treatment period and 46.0% [*n* = 23] during the ravulizumab treatment period), aplastic anemia (6.8% [*n* = 6] vs. 18.0% [*n* = 9], respectively), and BTH (4.5% [*n* = 4] vs. 8.0% [*n* = 4]) (Table 3).

**Table 3:** Transfusion patterns during the observation period

	Eculizumab treatment period (*N* = 80)	Ravulizumab treatment period (*N* = 80)
Patients who needed transfusion, n (%)	20 (25.0%)	15 (18.8%)
Total number of transfusions (days)	88	50
Number of packed red blood cell units transfused per patient	108	69
Reason for transfusion#, n (%)		
Aplastic anemia	6 (6.8%)	9 (18.0%)
BTH	4 (4.5%)	4 (8.0%)
Extravascular Hemolysis	12 (13.6%)	15 (30.0%)
Intravascular Hemolysis	42 (47.7%)	8 (16.0%)
Myelodysplastic syndromes	0 (0.0%)	3 (6.0%)
Other	22 (25.0%)	11 (22.0%)
Hemoglobin assessment (before transfusion) (g/dL)# - median (25th – 75th P)	7.9 (7.3–8.3)	7.7 (6.7–8.2)
Patients with stable hemoglobin* level in the absence of transfusion, n (%)	33 (41.3%)	54 (67.5%)

*BTH* = Breakthrough hemolysis; *P = *Percentile

# Percentages and statistics computed over the total number of transfusions, excluding missing values from the analysis

* Stable hemoglobin defined as < 2 g/dL decrease

Most patients (78; 97.5%) did not experience BTH during the observation period. A total of 7 BTH events occurred in 3 patients: 1 experiencing 4 BTH during the eculizumab treatment period, 1 experiencing 1 BTH during the ravulizumab treatment period, and 1 experiencing 1 BTH during both eculizumab and ravulizumab treatment. The median (25th – 75th P) LDH level across all BTH events during the eculizumab treatment period was 1175.0 (1151.0–1272.0) U/L and 924.0 (544.0–1304.0) U/L in the ravulizumab treatment period. Patients with BTH presented PNH-related signs or symptoms, such as anemia, fatigue, hemoglobinuria, dyspnea and erectile dysfunction. Overall, 85.7% (*n* = 6) of the BTH events required transfusion and 1 event led to hospitalization (Table 4). While a medical condition different to PNH triggered 1 over 5 events during the eculizumab treatment period, all BTH events were correlated to other medical conditions during the ravulizumab treatment period.

**Table 4:** Breakthrough hemolysis (BTH) events during the observation period

	Eculizumab treatment period (*N* = 80)	Ravulizumab treatment period (*N* = 80)
Number of BTH events per patient, n (%)		
0	78 (97.5%)	78 (97.5%)
1	1 (1.3%)	2 (2.5%)
4	1 (1.3%)	0 (0.0%)
Symptoms #§, n (%)		
Anemia (Hemoglobin < 10 g/dL)	5 (100.0%)	2 (100.0%)
Erectile dysfunction	0 (0.0%)	1 (50.0%)
Fatigue	3 (60.0%)	2 (100.0%)
Hemoglobinuria	1 (20.0%)	1 (50.0%)
Dyspnea	0 (0.0%)	1 (50.0%)
Other	0 (0.0%)	2 (100.0%)
* Jaundice*	–	1
* Fever*	–	1
Medical condition (trigger) correlated to the BTH §, n (%)		
None	4 (80.0%)	0 (0.0%)
Other	1 (20.0%)	2 (100.0%)
LDH (U/L) value - median (25th – 75th P)	1175.0 (1151.0–1272.0)	924.0 (544.0–1304.0)
Patient transfused §, n (%)		
Yes	4 (80.0%)	2 (100.0%)
Patient hospitalized §, n (%)		
No	5 (100.0%)	1 (50.0%)
Patient accessed the ER §, n (%)		
No	5 (100.0%)	2 (100.0%)

*BTH* = Breakthrough hemolysis; *ER = *Emergency room; *LDH = *Lactate dehydrogenase; *P = *Percentile

# More than one option could have been recorded

§ Analysis performed over the total number of breakthrough hemolysis events

Observing the patients’ QoL, the mean (SD) EORTC-QLQ-C30 global health status score was 69.1 (18.1) at baseline and 76.9 (17.0) at the 52-week follow-up visit, showing consistency during the observation period (Table 5). Similar results were observed in the five functional scales (physical, role, cognitive, emotional, and social) and the three symptom scales (fatigue, pain, and nausea and vomiting). Tables on other EORTC-QLQ-C30 scores are reported in the Online Resource 1. Overall, the mean (SD) FACIT-Fatigue score was constant for the whole observation period: 41.8 (6.0) at baseline and 40.4 (6.9) at the 52-week follow-up visit.

**Table 5:** EORTC QLQ-C30 scores during the observation period: global health status

	*N*	Mean (SD)	Median (25th – 75th *P*)	Range (min – max)
Global health status score				
Baseline	24	69.1 (18.1)	66.7 (50.0–83.3)	33.3–100.0
10 weeks follow-up visit	42	75.2 (18.7)	83.3 (66.7–83.3)	33.3–100.0
18 weeks follow-up visit	47	70.9 (19.6)	66.7 (66.7–83.3)	16.7–100.0
34 weeks follow-up visit	53	71.9 (19.4)	75.0 (66.7–83.3)	25.0–100.0
52 weeks follow-up visit	36	76.9 (17.0)	83.3 (66.7–87.5)	33.3–100.0

For each score, descriptives calculated over the number of patients included in the FULL ANALYSIS SET with usable questionnaire at the relevant time point. Questionnaires filled in after patient early withdrawal were not considered in the analysis. Each score ranges from 0 to 100; a higher score represents a higher (“better”) health status

**Fig. 1:**
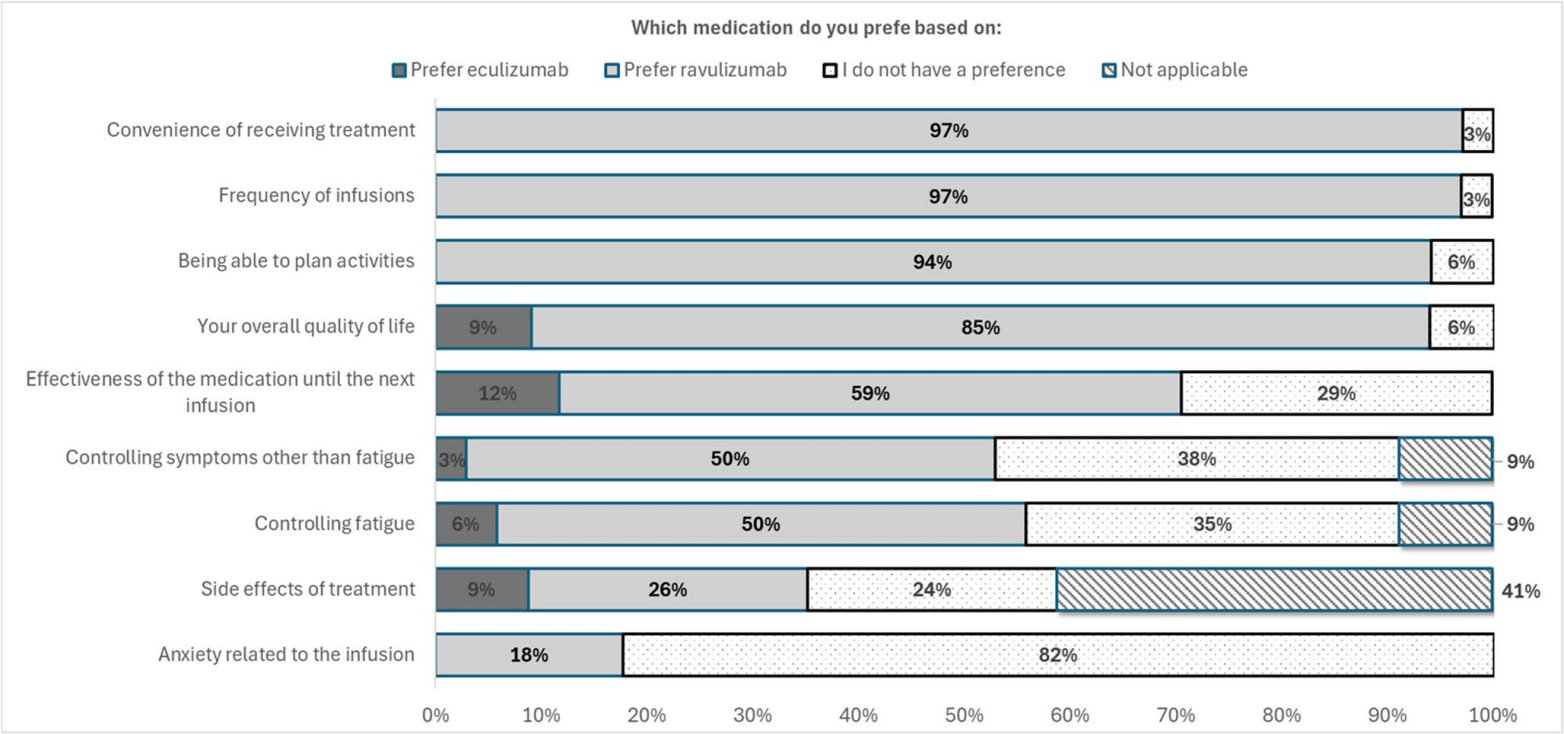
Factors driving patients’ treatment preferences according to the PNH-PPQ (*N* = 34)

Considering the patient’s preferences in terms of treatment among eculizumab and ravulizumab evaluated through the PNH-PPQ scale, 79.4% of the responders (27 out of 34) indicated an overall preference for ravulizumab. The main factors driving patients’ treatment preference were the convenience of receiving treatment, frequency of infusions, ability to plan activities and overall QoL (Fig. 1).

Overall, during the ravulizumab treatment period, 33.3% (*n* = 27) of patients in the Safety Set experienced at least one AE, mainly mild to moderate; serious AEs were observed in 4.9% (*n* = 4) of the patients. The most commonly reported AEs were general disorders and administration site conditions (9; 33.3%), including pyrexia and asthenia, and infections and infestations (9; 33.3%), mainly Covid-19. Headache, respiratory, thoracic and mediastinal disorders (mostly cough), gastrointestinal disorders, and musculoskeletal and connective tissue disorders were all reported by 4 patients (14.8%). Only one patient (3.7%) reported fatigue (Table 6). Among the patients with AEs, 7.4% (*n* = 6) patients had 8 events related to ravulizumab; in particular, headache (2; 7.4%); vertigo (1; 3,7%), chest pain (1; 3,7%), genital herpes (1; 3,7%), neutrophil count decreased (1; 3,7%), back pain (1; 3,7%), and pyelonephritis (1; 3,7%). Among the patients with serious AEs (4; 14.8% of all the AE), one event (pyelonephritis requiring hospitalization) was considered related to ravulizumab. Additionally, one life-threatening case of acute respiratory failure and pleural effusion and one hospitalization due to pericardial effusion have been reported. Two patients died during the observation study period, one of them due to an aggravated condition and the other one due to sarcoma. None of these fatal AEs have been considered treatment-related. No meningococcal infection and no thrombosis were reported.

**Table 6:** Description of adverse events reported by ≥ 5% of patients and all serious adverse events during the ravulizumab treatment period

System Organ Class - Preferred Term	Patients with AE *N* (%)	Patients with SAE *N* (%)
Any	27 (100.0%)	4 (14.8%)
Cardiac disorders	1 (3.7%)	1 (3.7%)
Pericardial effusion	1 (3.7%)	1 (3.7%)
Gastrointestinal disorders	4 (14.8%)	–
Anal abscess	1 (3.7%)	–
Gastroesophageal reflux disease	1 (3.7%)	–
Inguinal hernia	1 (3.7%)	–
Esophageal candidiasis	1 (3.7%)	–
General disorders and administration site conditions	9 (33.3%)	1 (3.7%)
Asthenia	2 (7.4%)	–
Chest pain	1 (3.7%)	–
Condition aggravated	1 (3.7%)	1 (3.7%)
Fatigue	1 (3.7%)	–
Pyrexia	6 (22.2%)	–
Hepatobiliary disorders	2 (7.4%)	–
Hepatomegaly	1 (3.7%)	–
Jaundice	1 (3.7%)	–
Infections and infestations	9 (33.3%)	–
Covid-19	4 (14.8%)	–
Gastrointestinal viral infection	1 (3.7%)	–
Genital herpes	1 (3.7%)	–
Nasopharyngitis	1 (3.7%)	–
Pharyngitis	1 (3.7%)	–
Respiratory syncytial virus infection	1 (3.7%)	–
Upper respiratory tract infection	1 (3.7%)	–
Musculoskeletal and connective tissue disorders	4 (14.8%)	–
Back pain	2 (7.4%)	–
Osteoporosis	1 (3.7%)	–
Pain in jaw	1 (3.7%)	–
Neoplasms benign, malignant and unspecified (incl. cysts and polyps)	2 (7.4%)	1 (3.7%)
Basal cell carcinoma	1 (3.7%)	–
Sarcoma	1 (3.7%)	1 (3.7%)
Nervous system disorders	4 (14.8%)	–
Headache	4 (14.8%)	–
Renal and urinary disorders	3 (11.1%)	1 (3.7%)
Chromaturia	1 (3.7%)	–
Hypercalciuria	1 (3.7%)	–
Pyelonephritis	1 (3.7%)	1 (3.7%)
Respiratory, thoracic and mediastinal disorders	4 (14.8%)	1 (3.7%)
Acute respiratory failure	1 (3.7%)	1 (3.7%)
Cough	2 (7.4%)	–
Oropharyngeal pain	1 (3.7%)	–
Pleural effusion	1 (3.7%)	1 (3.7%)
Skin and subcutaneous tissue disorders	2 (7.4%)	–
Eczema	1 (3.7%)	–
Erythema	1 (3.7%)	–
Vascular disorders	2 (7.4%)	–
Hematuria	1 (3.7%)	–
Presyncope	1 (3.7%)	–

Percentages computed over total number of patients with AE (*N* = 27)

## Discussion

The REACTION study showed that the real-world use of ravulizumab in patients previously treated with eculizumab was safe and effective, and switching from eculizumab to ravulizumab maintained hemolytic control, as demonstrated by the low incidence of BTH, the low proportion of patients needing transfusions and stable levels of LDH and hemoglobin. In addition, ravulizumab was the patient’s preferred choice of treatment, mainly for its convenience of administration.

The sample size of the cohort enrolled in the REACTION study (*N* = 81) is relevant for a rare disease. Patients presented with baseline characteristics similar to epidemiology data on PNH collected in the International PNH Registry [5]: most of the patients were of White/Caucasian ethnicity, with even distribution of males and females, and the mean (SD) age of the study population was 50.5 (16.1), with a median (25th – 75th P) history of disease of 9.0 (4.3–16.7) years.

At baseline, all the patients presented with clinically stable disease, which was expected considering previous treatment with eculizumab. Almost 70.0% (*n* = 55) of the patients presented with asymptomatic PNH, only 10.9% (*n* = 8) had LDH value ≥ 1.5 × ULN, indicating an effective control of intravascular hemolysis for most cases, and the median (25th – 75th P) hemoglobin level was 10.8 (9.5−11.9) g/dL, indicating control of anemia, which is a common complication of PNH [4]. Additionally, during prior treatment with eculizumab, 25.0% (*n* = 20) of patients needed transfusions, with 5.4 units per patient transfused, mainly for intravascular or extravascular hemolysis. These data mirrored the real-world data on patients with PNH, reporting a 50% reduction in transfusion rate among patients with transfusion history treated with eculizumab, passing from 10.6 red blood cell units per patient-year before eculizumab to 5.4 after 12 months from the start of eculizumab [15]. The switch from eculizumab to ravulizumab administered every 8 weeks showed consistency in disease control, with stability in all the relevant outcomes.

During the ravulizumab treatment period, the LDH normalization rate was maintained stable over time, confirming what was already observed during the ravulizumab clinical trials in a patient population previously treated with eculizumab [10, 13]. In particular, at the 52-week follow-up, 92.3% (*n* = 60) of the patients presented with LDH within the ULN or < 1.5 × ULN, in line with the proportion of patients who achieved the LDH ≤ 1.5 × ULN response threshold observed during the 2 years extension period of the ravulizumab clinical trials (94.7%) [13]. Further, the median (25th – 75th P) hemoglobin level at the 52-week follow-up (11.2 (9.9–12.5) g/dL) reflected the hemoglobin value observed during the 2-year extension study [13]. Also, the observed rate of transfusion avoidance was aligned with the one observed during the ravulizumab clinical trials [10, 13]. In the REACTION study, during the ravulizumab treatment period, 18.8% (*n* = 15) of the patients needed transfusions (14.4% in the 2-year extension [13]), with a lower rate compared to the one observed during the eculizumab treatment period (20; 25.0%). Additionally, the REACTION results suggested good outcome on transfusion avoidance, as among the 20 patients who received transfusion during the eculizumab period, 5 did not need transfusion after the switch to ravulizumab.

In patients with PNH receiving complement inhibitor therapy, the BTH represents a loss of disease control and can be associated with the return of the morbidity associated with PNH, including potentially life-threatening thromboembolic events [11]. During the REACTION study, 7 episodes of BTH were reported: 2 occurred during the ravulizumab treatment period and 5 BTH during the eculizumab treatment period. While only 1 out of 5 BTH during the eculizumab treatment period was triggered by a medical condition other than PNH, all BTH events during the ravulizumab treatment period were correlated to other medical conditions (i.e. infection and gastroenteritis), confirming that the longer half-life and complete and sustained terminal complement inhibition effect of ravulizumab considerably reduced pharmacokinetic BTH [2]. These real-world evidence are in line with evidences from registrational clinical trials of ravulizumab, involving both C5-inhibitor-naive patients and those with prior eculizumab experience. Long term data from these trials showed that during ravulizumab treatment there were few reported instances of BTH and that they were commonly associated with complement-amplifying conditions, whilst only a small percentage of events (1.8%) were associated with suboptimal inhibition of C5 (i.e. serum free C5 ≥ 0.5 µg/mL). In addition, the reported breakthrough-intravascular hemolysis events were generally associated with a lower excursion of LDH level (2–3 × ULN), not associated with thromboembolic events, and did not require modification, interruption, or withdrawal of ravulizumab treatment [7].

Finally, the QoL assessment of the patients enrolled in the REACTION study resulted from the EORTC Global Health/QoL and the FACIT-Fatigue scores, widely used and well-studied validated scales in PNH, even if were developed for assessing the QoL of cancer patients [6, 16, 17].

During the study observation period, the mean (SD) EORTC-QLQ-C30 global health status score was aligned with the one of the general adult population (75.5 [6]), being 69.1 (18.1) at baseline and 76.9 (17.0) at the 52-week follow-up visit, without any clinically meaningful change (i.e. improvement of ≥ 10 points [18]) before and after the switch from eculizumab to ravulizumab.

Fatigue is the most common symptom associated with PNH, and an increase in FACIT-F scores signifies a reduction in the impact of fatigue on the patient’s quality of life [4, 19]. In patients with untreated PNH, the median FACIT-F score is 34.0, compared to 43.6 in the general adult population [6, 17]. Observing the patients’ responses during the REACTION study, the mean (SD) FACIT-Fatigue score was stable for the whole observation period, being 41.8 (6.0) at baseline and 40.4 (6.9) at the 52-week follow-up visit and was aligned with the one of the general adult population.

The PNH-PPQ is a Patient-Reported Outcome (PRO) measure developed to capture patients’ overall treatment preference based on key aspects of PNH treatment, including symptom management, infusion frequency, and overall burden of treatment [20, 21]. Considering the patient’s preference in the REACTION study, most of them indicated an overall preference for ravulizumab compared to eculizumab, mainly for frequency of infusions, the convenience of receiving treatment, ability to plan activities and overall QoL, confirming the results already collected by an extension of ravulizumab clinical trials and highlighting the importance of these key factors for patients with PNH in a real-world setting [20].

In terms of tolerability, the switch from eculizumab to ravulizumab was safe, with 33.3% of patients experiencing at least one AE, and only 4.9% of patients having a serious AE, showing a safety profile more favorable than the one observed in the clinical trials and confirming the observation that, in general, the incidence of AEs appears to decrease over time [18].

The interpretation of the REACTION study results should consider the intrinsic limitations of an observational study, which were accounted for in the study design. In particular, sites were not randomly sampled from the whole pool of Italian clinics but were selected according to their experience in PNH patient management and to ensure a sufficient number of patients was enrolled in the study, given the rareness of the disease. Site selection reflects the clinical practice in Italy. Despite these limitations, the study provided a wide real-world description of the current Italian clinical practice of treatment with ravulizumab in PNH patients.

## Conclusion

The REACTION study’s results showed in an Italian real-world setting that switching patients with PNH from eculizumab to ravulizumab is effective and safe, confirming what has already been shown in phase III clinical trials. Ravulizumab effectively maintained control of terminal complement activity and intravascular hemolysis and sustained stable levels of LDH and hemoglobin throughout the observation period. The transfusion needs and the total number of BTH events were lower during the ravulizumab treatment period than during the eculizumab treatment period. Furthermore, during ravulizumab, both recorded BTH events were triggered by other medical conditions, suggesting the reduction of pharmacokinetic BTH. This was further supported by the observed trend of a low LDH levels during BTH episodes with ravulizumab compared to eculizumab. Finally, despite both eculizumab and ravulizumab increased patients’ quality of life, the longer half-life of ravulizumab considerably improved patient convenience with a benefit on patient-reported outcomes as measures of QoL.

## Supplementary Information

Below is the link to the electronic supplementary material.

Supplementary Material 1

## Data Availability

The data that support the findings of this study are available from the corresponding author upon reasonable request.
