# Supplementary material for: Real life use of ravulizumab in Italian patients with paroxysmal nocturnal hemoglobinuria: evidence from the REACTION observational study
Source: Ann Hematol. 2026 Jan 22;105(2):50. doi: 10.1007/s00277-026-06792-w (PMC12823630; doi:10.1007/s00277-026-06792-w)
Supplement: Supplementary file 1 — Supplementary Material 1 [file 277_2026_6792_MOESM1_ESM.docx]

**Real life use of ravulizumab in Italian patients with paroxysmal nocturnal hemoglobinuria: evidence from the REACTION observational study**

**Authors**

Anna Paola Iori^1^, Antonio De Vivo^2^, Eros Di Bona^3^, Giovanni Caocci^4^, Francesca Fioritoni^5^, Fabio Ciceri^6^, Eloise Beggiato^7^, Davide Rapezzi^8^, Angela Amendola^9^, Amalia Figuera^10^, Carmine Selleri^11^, Francesco Longu^12^, Bruno Fattizzo^13^, Alessandra Tucci^14^, Alessandro Cignetti^15^, Valeria Amico^16^, Simona Sica^17^, Elisabetta Metafuni^18^, Simona Raso^19^, Tiziana Anna Urbano^20^, Luana Marano^21^, Nicola Di Renzo^22^, Pierangelo Spedini^23^, Alessandro Rambaldi^24^, Francesco Lanza^25^, Cristina Clissa^26^, Cristina Danesin^27^, Maria Bruna Greve^28^, Sergio Cabibbo^29^, Alessandra Ori^30^, Francesca Cassanelli^30^, Federica Sottana^31^, Benedetta Campolo^32^, Giulia Gasparri^31^, Fabio Carini^31^, and Wilma Barcellini^33^

1. A.O.U. Policlinico Umberto I-Ematologia, Rome, Italy, iori@bce.uniroma1.it
2. A.O.U. Policlinico Sant'Orsola-UOC Ematologia, Bologna, Italy
3. Ospedale San Bassiano-Oncoematologia, Bassano del Grappa (Vicenza), Italy
4. Ospedale Businco-Ematologia e CTMO, Cagliari, Italy
5. Ospedale Santo Spirito-UOC Ematologia, Pescara, Italy
6. Università Vita-Salute San Raffaele, Milan Italy

IRCCS Ospedale San Raffaele, Milan, Italy

1. A.O.U. Città della Salute e della Scienza-Ematologia, Turin, Italy
2. ASO Santa Croce e Carle-Ematologia, Cuneo, Italy
3. AOR SAN CARLO, Dipartimento Oncologico, UOC Ematologia con Centro trapianto di Midollo, Cellule staminali e terapie cellulari, Potenza, Italy
4. A.O.U. Policlinico Rodolico-San Marco-Ematologia, Catania, Italy
5. A.O.U. S.Giovanni di Dio e R.D'Aragona-UOC Ematologia e Trapianti Cellule Staminali Emopoietiche, Salerno, Italy
6. A.O.U. Sassari-Ematologia, Sassari, Italy
7. Fondazione IRCCS Ca' Granda Ospedale Maggiore Policlinico-SC Ematologia, Milan, Italy
   Department of Oncology and Hemato-Oncology, University of Milan, Milan, Italy
8. ASST Spedali Civili-Ematologia, Brescia, Italy
9. A.O. Ordine Mauriziano-SCDU Ematologia e Terapie cellulari, Turin, Italy
10. Azienda Ospedaliera San Pio-Servizio di Immunoematologia, Benevento, Italy
11. Fondazione P.U. A.Gemelli IRCCS-UOC Ematologia e Trapianto di cellule staminali emopoietiche, Rome – Università Cattolica Sacro Cuore, Italy
12. Fondazione P.U. A.Gemelli IRCCS-UOC Ematologia e Trapianto di cellule staminali emopoietiche, Rome, Italy
13. A.O.O.R. Villa Sofia Cervello P.O. Cervello-UOC di Ematologia per le Malattie Rare del Sangue e degli Organi Ematopoietici, Palermo, Italy
14. Ospedale San G. Moscati-SC Ematologia, Taranto, Italy
15. Ospedale San G. Moscati-Ematologia, Avellino, Italy
16. Ospedale V. Fazzi-UOC Ematologia, Lecce, Italy
17. Ospedale di Cremona-Ematologia, Cremona, Italy
18. ASST Papa Giovanni XXIII-UOC Ematologia, Bergamo, Italy
19. Università di Bologna - Ospedale S. Maria delle Croci-UOC Ematologia, Ravenna, Italy
20. A.O.U.I. Verona Ospedale Borgo Roma-UOC Ematologia e Centro Trapianti di Midollo Osseo, Verona, Italy
21. Ospedale Ca' Foncello-Ematologia, Treviso, Italy
22. GOM Bianchi Melacrino Morelli-Ematologia, Reggio Calabria, Italy
23. Ospedale "Giovanni Paolo II"-UOSD Ematologia, Ragusa, Italy
24. IQVIA Solutions Italy S.r.l., Modena, Italy
25. Alexion Pharma Italy, Milan, Italy
26. Alexion Pharma, Barcelona, Spain
27. Fondazione IRCCS Ca' Granda Ospedale Maggiore Policlinico-SC Ematologia, Milan, Italy

**Online Resource 1**

***EORTC-QLQ-C30*** ***scores during the observation period***

**Table 7. EORTC QLQ-C30 scores during the observation period: five functional scales (physical, role, cognitive, emotional, and social)**

|  | **N** | **Mean (SD)** | **Median**  **(25^th^ – 75^th^ P)** | **Range**  **(min – max)** |
| --- | --- | --- | --- | --- |
| Physical Functioning score |  |  |  |  |
| Baseline | 24 | 80.8 (17.4) | 86.7 (70.0 – 93.3) | 40.0 – 100.0 |
| 52 weeks follow-up visit | 36 | 84.4 (14.9) | 86.7 (76.7 – 96.7) | 46.7 – 100.0 |
| Role Functioning score |  |  |  |  |
| Baseline | 24 | 84.0 (21.1) | 100.0 (66.7 – 100.0) | 33.3 – 100.0 |
| 52 weeks follow-up visit | 36 | 80.6 (25.0) | 91.7 (66.7 – 100.0) | 0.0 – 100.0 |
| Emotional Functioning score |  |  |  |  |
| Baseline | 24 | 79.2 (23.1) | 83.3 (66.7 – 100.0) | 16.7 – 100.0 |
| 52 weeks follow-up visit | 36 | 78.5 (18.9) | 83.3 (66.7 – 95.8) | 33.3 – 100.0 |
| Cognitive Functioning score |  |  |  |  |
| Baseline | 24 | 84.0 (15.1) | 83.3 (66.7 – 100.0) | 50.0 – 100.0 |
| 52 weeks follow-up visit | 36 | 84.3 (18.7) | 91.7 (66.7 – 100.0) | 33.3 – 100.0 |
| Social Functioning score |  |  |  |  |
| Baseline | 24 | 79.2 (19.8) | 83.3 (66.7 – 100.0) | 33.3 – 100.0 |
| 52 weeks follow-up visit | 36 | 85.6 (19.2) | 100.0 (66.7 – 100.0) | 16.7 – 100.0 |

For each score, descriptives calculated over the number of patients included in the FULL ANALYSIS SET with usable questionnaire at the relevant time point. Questionnaires filled in after patient early withdrawal were not considered in the analysis. Each score ranges from 0 to 100; a higher score represents a higher ("better") health status.

**Table 8. EORTC QLQ-C30 scores during the observation period: three symptom scales (fatigue, pain, and nausea and vomiting)**

|  | **N** | **Mean (SD)** | **Median**  **(25^th^ – 75^th^ P)** | **Range**  **(min – max)** |
| --- | --- | --- | --- | --- |
| Fatigue score |  |  |  |  |
| Baseline | 24 | 31.5 (22.1) | 33.3 (16.7 – 44.4) | 0.0 – 77.8 |
| 52 weeks follow-up visit | 36 | 31.8 (21.6) | 33.3 (11.1 – 44.4) | 0.0 – 77.8 |
| Nausea and vomiting score |  |  |  |  |
| Baseline | 24 | 6.9 (14.7) | 0.0 (0.0 – 8.3) | 0.0 – 50.0 |
| 52 weeks follow-up visit | 36 | 3.7 (7.0) | 0.0 (0.0 – 0.0) | 0.0 – 16.7 |
| Pain score |  |  |  |  |
| Baseline | 24 | 2.1 (5.6) | 0.0 (0.0 – 0.0) | 0.0 – 16.7 |
| 52 weeks follow-up visit | 36 | 12.0 (19.8) | 0.0 (0.0 – 16.7) | 0.0 – 83.3 |

For each score, descriptives calculated over the number of patients included in the FULL ANALYSIS SET with usable questionnaire at the relevant time point. Questionnaires filled in after patient early withdrawal were not considered in the analysis.

Note. Each score ranges from 0 to 100; a higher score represents a higher ("worse") level of symptoms.

**Table 9. EORTC QLQ-C30 scores during the observation period: additional symptoms (dyspnoea, loss of appetite, insomnia, constipation and diarrhoea)**

|  | **N** | **Mean (SD)** | **Median**  **(25^th^ – 75^th^ P)** | **Range**  **(min – max)** |
| --- | --- | --- | --- | --- |
| Dyspnoea score |  |  |  |  |
| Baseline | 24 | 19.4 (23.9) | 16.7 (0.0 – 33.3) | 0.0 – 100.0 |
| 52 weeks follow-up visit | 36 | 12.0 (18.1) | 0.0 (0.0 – 33.3) | 0.0 – 66.7 |
| Insomnia score |  |  |  |  |
| Baseline | 24 | 16.7 (27.8) | 0.0 (0.0 – 33.3) | 0.0 – 100.0 |
| 52 weeks follow-up visit | 36 | 19.4 (21.6) | 16.7 (0.0 – 33.3) | 0.0 – 66.7 |
| Appetite loss score |  |  |  |  |
| Baseline | 24 | 4.2 (11.3) | 0.0 (0.0 – 0.0) | 0.0 – 33.3 |
| 52 weeks follow-up visit | 36 | 5.6 (12.6) | 0.0 (0.0 – 0.0) | 0.0 – 33.3 |
| Constipation score |  |  |  |  |
| Baseline | 24 | 8.3 (17.7) | 0.0 (0.0 – 0.0) | 0.0 – 66.7 |
| 52 weeks follow-up visit | 35 | 10.5 (19.4) | 0.0 (0.0 – 33.3) | 0.0 – 66.7 |
| Diarrhoea score |  |  |  |  |
| Baseline | 24 | 6.9 (13.8) | 0.0 (0.0 – 0.0) | 0.0 – 33.3 |
| 52 weeks follow-up visit | 35 | 8.6 (16.8) | 0.0 (0.0 – 0.0) | 0.0 – 66.7 |

For each score, descriptives calculated over the number of patients included in the FULL ANALYSIS SET with usable questionnaire at the relevant time point. Questionnaires filled in after patient early withdrawal were not considered in the analysis. Each score ranges from 0 to 100; a higher score represents a higher ("worse") level of symptoms.

**Table 10. EORTC QLQ-C30 scores during the observation period: Financial Difficulties**

|  | **N** | **Mean (SD)** | **Median**  **(25^th^ – 75^th^ P)** | **Range**  **(min – max)** |
| --- | --- | --- | --- | --- |
| Financial difficulties score |  |  |  |  |
| Baseline | 24 | 18.1 (29.5) | 0.0 (0.0 – 33.3) | 0.0 – 100.0 |
| 52 weeks follow-up visit | 35 | 10.5 (26.5) | 0.0 (0.0 – 0.0) | 0.0 – 100.0 |

For each score, descriptives calculated over the number of patients included in the FULL ANALYSIS SET with usable questionnaire at the relevant time point. Questionnaires filled in after patient early withdrawal were not considered in the analysis. Each score ranges from 0 to 100; a higher score represents a higher ("worse") level of symptoms.
